# Perceived Barriers to Participation in Clinical Research Amongst Trauma and Orthopaedic Community: A Survey of 148 Consultants and Junior Doctors in Wales

**DOI:** 10.7759/cureus.19694

**Published:** 2021-11-18

**Authors:** Aurelia Vas, Prashanth D'sa, Hamid Daud, Avadhut Kulkarni, Stefan Bajada, Eleanor C Carpenter

**Affiliations:** 1 General Surgery, Princess of Wales Hospital, Bridgend, GBR; 2 Trauma and Orthopaedics, University Hospital of Wales, Cardiff, GBR; 3 Trauma and Orthopaedics, Withybush General Hospital, Haverfordwest, GBR; 4 Trauma and Orthopaedics, Glangwili General Hospital, Carmarthen, GBR

**Keywords:** junior doctors, consultants, trauma & orthopaedics, barriers, clinical research

## Abstract

Background: Research has led to substantial improvement in health and quality of life. It is pertinent for doctors to participate in research to keep up with the advances of modern medicine and forms one of the seven pillars of clinical governance defined by the General Medical Council. However, clinicians face multiple barriers to participating in research. The objective of this study was to identify barriers in participation and to recommend solutions for better engagement in orthopaedic research.

Methodology: Trauma and Orthopaedic consultants and junior doctors in Wales were asked to complete a web-based survey with 15 questions about barriers to participation and suggestions for increasing involvement in clinical research.

Results: A total of 148 completed forms were received which included 60 consultants and 88 junior doctors. The response rate was 86%. The most frequently reported barriers to clinical research were time constraints, excess paperwork, lack of knowledge about research methods, and lack of awareness of ongoing research studies. Most participants were keen to be involved in research in the future. Majority responded that they would more likely take part in research activity if there were formal training sessions and more dedicated research sessions scheduled into their timetable. Need for more incentives and allocation of a research officer were other suggestions. Most orthopaedic staff recognised the relevance of research to their job/training.

Conclusion: There are multiple perceived barriers to participating in research at all levels in the orthopaedic community; however, these could be mitigated by implementing simple measures.

## Introduction

High-quality research forms the basis of evidence-based medicine. Research helps derive new knowledge aimed at discovering best practices in a systematic approach. We all know the huge benefits research has had for the health and quality of life of our patients in the last century. There has been a dramatic increase in clinical research projects in recent decades due to the rapid development of new drugs, treatments, and devices. These have added new challenges in the form of needing greater participation by physicians and patients and in fulfilling these in a stipulated timescale for a meaningful outcome. Research is highlighted as the key part of the National Health Service (NHS, UK) constitution, guiding trusts to be more cost-effective by recognizing what is not working, supporting the decommissioning of interventions, and better tailoring services to meet the needs of patients [1].

A strong grasp of the fundamental principles of research and its applications to clinical practice is essential for all doctors. Research is featured in the General Medical Council’s (GMC) good medical practice guidance [2], generic professional capabilities framework [3], the independent shape of training review [4], and the Royal College of Surgeons (RCS) standards for good surgical practice [5]. Furthermore, evidence of participation in research is an essential part of certification guidelines for various surgical specialties, as set by Joint Committee on Surgical Training (JCST) [6]. Trauma and Orthopaedic (T&O) specialty trainees are expected to demonstrate engagement with and commitment to evidence-based practice through various research-related activities [6]. These include reflections from journal club/literature review, presentation or publication of research, recruiting participants for studies, completion of a higher degree, membership of research collaborative network, or National Institute for Health Research (NIHR) groups, contribution to a research grant, and ethics committee applications [6]. Research training embedded within clinical training programmes has been shown to promote trainees’ critical thinking, analysis, improve the quality of patient care, and facilitate post-training academic output [7].

There is a growing demand for participation by consultant surgeons and trainees in various research studies in T&O. There is no published literature to quantify the interest and perceived barriers for participation in clinical research activity in the T&O community. The survey aimed to identify the current level of interest and barriers that are experienced by Welsh T&O consultants and junior doctors (JD) to participate in clinical research and recognise measures to improve their engagement. We hypothesised that there is a lot of interest amongst surgeons at every level to participate in clinical research, yet there are multiple barriers preventing them to do so. Therefore, we hope to suggest solutions to improve this.

## Materials and methods

A web-based survey using google forms was designed by the authors. The study was approved by the T&O research department as a service evaluation and did not require ethical committee approval. The questions on barriers and solutions were templated based on previous research in this area in other specialties [8-18]. This was initially piloted amongst junior doctors and consultants in the senior author’s hospital. Data and feedback from this were used to create the final questions of the survey by clarifying structure and wording. A free text section at the end of the survey was included for the consultants based on pilot survey feedback. The main survey included three sections, with 10 questions about barriers to participation in research activity, four questions to assess what would encourage them to take part in research, and a final question on their perception of the relevance of research participation to their job in the NHS. The questions are tabulated in Table 1. Responses to all questions were kept mandatory and were collected in the form of five-point Likert scale (strongly disagree to strongly agree). 

**Table 1:** The questionnaire SD: strongly disagree, D: disagree, N: neutral, A: agree, SA: strongly agree, NHS: National Health Service.

Section 1: The barriers	Responses in SD/D/N/A/SA
I am not aware of any research studies going on.	
I am not familiar with the procedure to take part in research studies.	
I have time constraints.	
I don't have confidence to approach the patients for taking part in studies.	
The work involved is too challenging.	
There is too much paperwork.	
It is not worth the effort.	
I am not interested in doing research work.	
There are no rewards/recognition associated with research work.	
I feel it may have negative impact on doctor-patient relationship.	
Section 2: The solutions	
What would encourage more surgeons to take part in research studies?	
If there is research officer who could motivate/guide me to get into research.	
If there are any training sessions that would help me to join the research studies.	
There should be more dedicated research sessions.	
There should be more rewards/incentives for recruiting patients into research studies.	
Section 3: Relevance to your job in the NHS	
Do you think it is relevant for you to participate in research in your role/training?	

The survey weblink was distributed by the authors via email and private social media text using qualitative purposive convenience sampling technique to T&O junior doctors and consultants in Wales, UK. The survey was kept open for two months, with non-responders being sent one reminder after two weeks in the study period. Results were analyzed and summarized using simple descriptive statistical methods in two groups, one for consultants and one for junior doctors. Junior doctors included foundation year, core surgical trainees, and equivalent grades which are also commonly termed as senior house officers in the UK, specialty T&O trainees/specialty doctor/staff grades, who are commonly termed as registrars. Free text comments from consultants were not subject to statistical analysis and are listed in Table 2.

**Table 2:** Free text comments and suggestions by consultants

Responses
1. Most of us want to participate but lack the time, resources, and incentives to do so, such are the pressures of day-to-day orthopaedic practice.
2. The lack of clear guidance and assistance by local trusts in research is one of the biggest obstacles I have faced.
3. The Health Board actively discourages research by their actions.
4. One of the main constraints to doing proper research projects is infrastructure. Departments with research nurses who can recruit patients and paperwork would help a lot.
5. The greatest constraint to research at senior levels is time. As a result, too many irrelevant research projects are carried out which does not contribute to improvement in patient care. Centralized dedicated research units should be tasked to educate and train trainees, and consultants interested in research should seek honorary academic positions.
6. Lack of organized research meetings.
7. 98% of consultants in Wales are not lecturers or professors and hence don't get paid to do any research. Time spent on any research activity is thus almost always at the cost of some other work or personal time.
8. There is no protected time to do research, and any grants/applications for dedicated research time are looked at badly and are disfavoured by trust/management.
9. Need better access to support research staff, to assist with recruiting in busy clinics.
10. No recognition at all for consultants producing research in their own time over and above clinical commitments.

## Results

**Figure 1:**
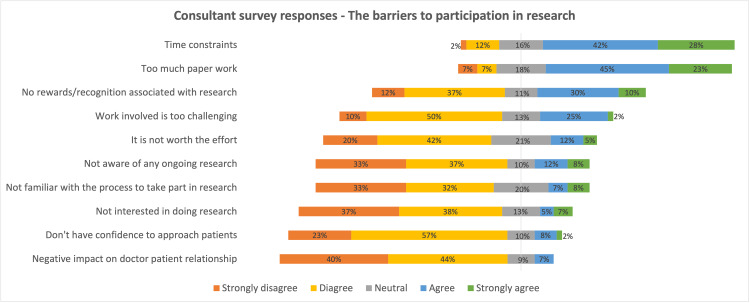
Diverging stacked bar chart summarizing the survey response on perceived barriers from consultants

**Figure 2:**
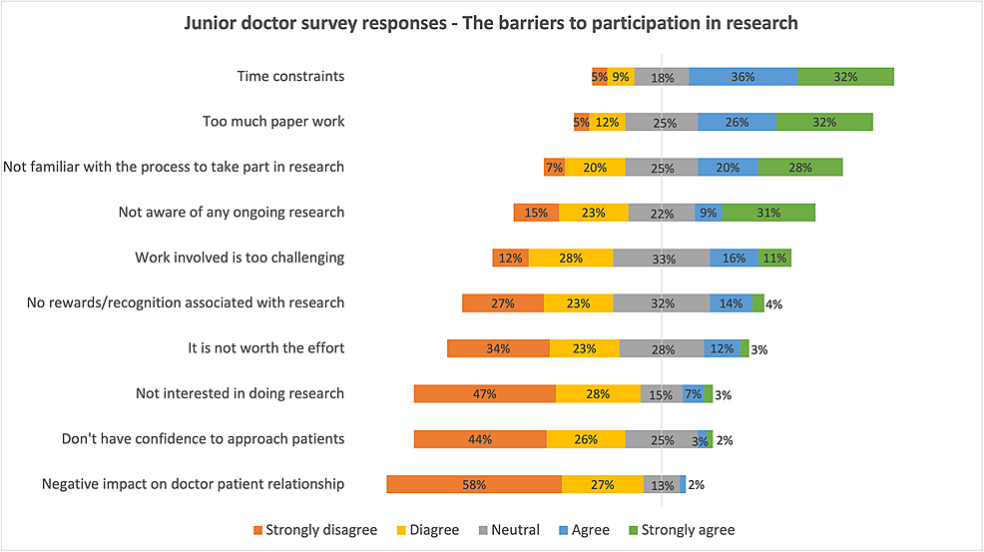
Diverging stacked bar chart summarizing the survey response on perceived barriers from junior doctors

A total of 148 completed forms were received: a response rate (RR) of 86%, which included 60 consultants (RR, 80%) and 88 JD (59 registrar grade: RR, 91%; and 29 senior house officer grade: RR, 88%). The most frequently reported barriers to clinical research by both tiers were time constraints, excessive paperwork involved, unfamiliarity with the participation process, and unawareness of any ongoing research studies. The responses in the Likert scale are detailed in diverging stacked bar charts below (Figures 1, 2).

**Figure 3:**
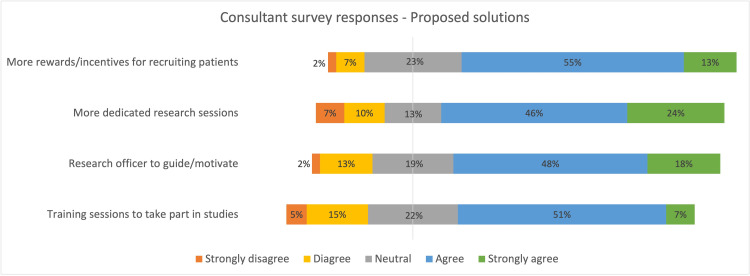
Diverging stacked bar chart summarizing the survey response on proposed solutions from consultants

**Figure 4:**
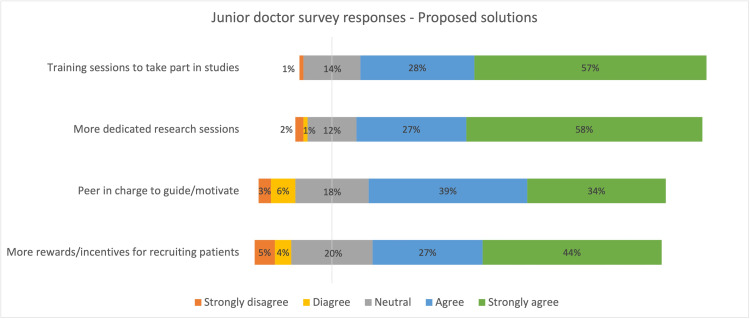
Diverging stacked bar chart summarizing the survey response on proposed solutions from junior doctors

**Figure 5:**
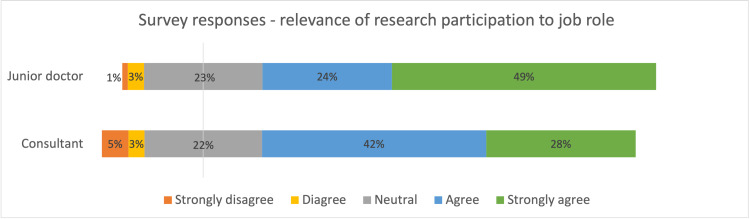
Diverging stacked bar chart summarizing survey response on relevance of research participation to job role

The majority indicated that they would more likely participate in research if there were formal training sessions, more dedicated research sessions scheduled into their timetable, more rewards/recognition for participation, and if there was a peer/research officer who could motivate and guide them (Figures 3, 4). The majority agreed that research participation is relevant for their job role and training in the NHS (Figure 5).

## Discussion

This survey details the current academic atmosphere within Trauma and Orthopaedic department in a large region within the NHS and the barriers for clinical research involvement perceived by the current and the future leaders in this specialty. Multiple studies have investigated the barriers faced by physicians, trainees, and nurses in other specialities to participate in clinical research and have suggested specific solutions tailored to them [8-18]. However, there have been no published studies that have looked specifically into the hurdles faced by Trauma and Orthopaedic consultant surgeons (C) and junior doctors.

The main findings from the survey data indicate a strong appetite for engaging in research activities through all tiers of this surgical subspecialty (75%). In terms of perceived barriers, top-ranked themes were time constraints (strongly agree/agree (SA/A) = C: 70%, JD: 68%), and the amount of paperwork involved (C: 68%, JD: 58%), followed by other frequent hurdles such as lack of rewards/recognition (C: 40%, JD: 18%), research being perceived as challenging work (C: 27%, JD: 27%), work being not worth the effort (C: 17%, JD: 15%), unawareness of any ongoing projects (C: 20%, JD: 40%), and lack of interest (C: 12%, JD: 10%) (Figures 1, 2). These have been identified as common themes in studies by other specialities across the globe in various proportions [8-18]. These are all challenges in themselves to conquer, due to busy clinical settings, inadequate staffing levels, and lack of research support staff. This survey gives useful information as to how we might help promote research participation among T&O junior doctors and senior surgeons. However, as mentioned by Mustafa et al. [16], “a good balance of academic culture that promotes research but also recognizes individual interests and career aspirations will be most fruitful in supporting those with an active interest in research and providing them with an encouraging environment to flourish in”.

Personal disinterest in research as a barrier is well described in the literature. In our survey, a minority of participants (12% C, 10% JD) responded as not being interested in participating in any form of research. These figures are quite low in comparison to the surveys amongst other subspecialties mentioned in the literature (18% by Olaussen et al., 20% by Al-Taha et al., 22% by Myint et al., 32% by Hames et al.), suggesting a relatively strong appetite for clinical research amongst T&O surgeons in Wales [9,11,12,15].

The majority of participants agreed upon the need for better rewards/recognition associated with participation in research work, protected research sessions built into their weekly timetables, organised regular training sessions on available research projects, and mentorship in the form of a peer or research officers to motivate and guide (Figures 3, 4). We believe these are the areas where we could target our interventions to improve participation in clinical research.

Since the presentation of the findings from the survey at the senior author’s centre, the following changes have been made to improve participation by trainees in clinical research: All junior doctors are allocated protected research time on their weekly rota, provided adequate staffing levels can be maintained. Training sessions on available research trials and support/guidance for those interested in taking part in these have been organised by the research department monthly and have been received with great feedback. Interested junior doctors have been supported and guided to apply for the associate principal investigator scheme run by the NIHR [19], who in turn will be guiding/motivating other junior doctors to take part in trials, coordinating recruitment, and day-to-day running of the trial along with the research team. This will also aid in improving trainees’ leadership and management capabilities, a great addition to the portfolio, and a potential step towards setting up of own trials in the future. Evidence from our centre, on the role of trainee research advocate for a trauma randomised control trial, has shown significant improvement in recruitment and interest by peers to join the trials [20].

The strengths of this study include responses from all tiers of doctors of a surgical specialty and relatively large numbers spread across a large geographical location covering all types of hospitals from a major trauma centre to a district general hospital. Limitations, however, include sampling bias (not everyone working in these hospitals was invited for the survey due to purposive convenience sampling technique), the inherent bias of survey technique, and the absence of focus groups, interviews, and qualitative methods. A better view of the hurdles faced would be achieved if surgeons from throughout the UK were to take part in the study; however, this study covers the largest geographical location in the UK, and there are no compelling reasons for it not to be reflective of a wider population.

To the best of our knowledge, this is the first study diving into the barriers faced by the Trauma and Orthopaedic community, understanding the extent to which these affect our surgeons, and can thus aid us in offering some simple solutions to overcome these hurdles. We recommend three areas for further improvement, which include incorporating dedicated/protected research times on the weekly timetable/rota for both consultant and junior doctors, identifying junior doctors with a special research interest to motivate/guide other colleagues along with research officers where available, and organising regular training sessions on available projects to guide participation in research trials.

## Conclusions

It is more important than ever that all clinicians take part in research to further aid in the advancement of medicine and healthcare in the new century. Multiple barriers are faced for participating in research at all levels in the orthopaedic community; however, these could be mitigated by implementing some simple steps. We suggest incorporating protected research times, identifying peers with a special interest in research, and regular training sessions on available projects to improve participation in research activity.
